# Management of New Onset Amphetamine-Induced Acute Decompensated Heart Failure in a Young Adult: Case Report

**DOI:** 10.7759/cureus.43854

**Published:** 2023-08-21

**Authors:** Yuli Breier, Eli Azrak, Wesley Banks, Robert Kushmakov, Irina Aleshinskaya

**Affiliations:** 1 Surgery, Touro College of Osteopathic Medicine, New York City, USA; 2 Emergency Medicine, Touro College of Osteopathic Medicine, New York City, USA; 3 Emergency Medicine, Staten Island University Hospital, New York City, USA

**Keywords:** drug and substance abuse, chronic drug use, decompensated heart failure, congestive heart faiulre, methamphetamine induced cardiomyopathy, dilated cardiomyopathy

## Abstract

Amphetamine-induced heart failure is a well-recognized complication of chronic amphetamine abuse. Although the exact mechanisms underlying the development of this condition are not fully understood, it is believed to be due to a combination of direct toxicity to the myocardium, increased sympathetic activity, and oxidative stress. Amphetamine-induced heart failure typically presents with symptoms such as dyspnea, fatigue, and peripheral edema and can lead to significant morbidity and mortality. Diagnosis is based on a combination of clinical and laboratory findings, including echocardiography and cardiac biomarkers. Treatment typically involves cessation of amphetamine use, management of heart failure symptoms, and aggressive medical therapy with agents such as beta-blockers and angiotensin-converting enzyme (ACE) inhibitors. However, the long-term prognosis for patients with amphetamine-induced heart failure remains poor, highlighting the need for increased awareness and prevention efforts surrounding this growing public health concern. In this case, a 21-year-old male presented to the emergency department (ED) with acute-onset decompensated heart failure due to amphetamine abuse.

## Introduction

Illegal drug use is no stranger to our generation, including within the advanced, developed world of the United States of America (USA). The prevalence of illegal drug use in the USA was estimated to be 13.0% in people above the age of 12 years old in 2019 and 0.6% in people above the age of 12 years old reporting a methamphetamine use disorder in 2020 (approximately 1.5 million people) [1,2]. The average duration of methamphetamine use before a diagnosis of congestive heart failure is five years, with almost half (18%) of the reported diagnoses made in the first 12 months. Congestive heart failure was diagnosed in some cases even after a single use [3]. 

Methamphetamine use induces potent vasoconstriction that can result in severe vasospasm of the coronary arteries and microvasculature, resulting in myocardial ischemia. In the heart, methamphetamine promotes myocardial structural and electrical remodeling, which may promote cardiac arrhythmias. Ultimately, methamphetamine induces profound mitochondrial dysfunction and cardiac myocyte death, driving dilated cardiomyopathy and heart failure [4]. 

Guidelines for the treatment of methamphetamine-associated cardiomyopathy have not been established. Short-term management should be inferred from the symptomatic treatments for forms of acute heart failure. Currently, no studies have evaluated long-term medical therapies, specifically in patients with methamphetamine-associated cardiomyopathy. Patients with methamphetamine-associated cardiomyopathy should be treated with guideline‐directed medical therapy for heart failure with reduced ejection fraction [4]. An important feature of methamphetamine-associated cardiomyopathy is the potential for significant recovery of cardiac function with cessation of methamphetamine use. Discontinuation of methamphetamine use was associated with improvements in heart failure symptoms and cardiac function. Consideration of a primary prevention implantable cardioverter-defibrillator should follow the guidelines for heart failure with reduced ejection fraction. Since cardiac function can improve after drug cessation, left ventricular function should be reassessed after abstinence, and the placement of an implantable cardioverter-defibrillator is generally not necessary. Clinical focus should be placed on patient rehabilitation for complete cessation of methamphetamine use [5].

In this case report, we present a 21-year-old patient with a history of eight years of use who presented to the emergency department (ED) with severe shortness of breath and was diagnosed with CHF with an ejection fraction of less than 20%.

## Case presentation

**Figure 1:**
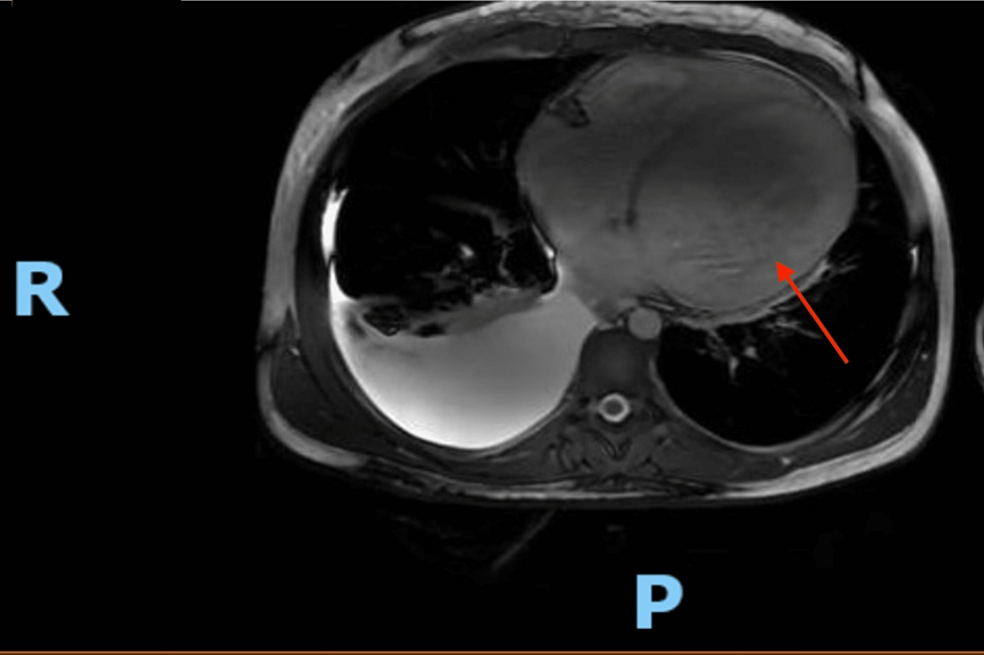
MRI of methamphetamine-induced cardiomyopathy This MRI shows the extensively dilated ventricles in this case. The left ventricle (marked by a red arrow) is remarkably dilated. The study was taken during an episode of noteworthy acute decompensated CHF and demonstrates the magnitude of the pathology. Massive right-sided posterior pleural effusion is noteworthy regarding the crescent shaped fluid collection in the right/posterior chest cavity (marked as R for right side and P for posterior).

In this case, a 21-year-old male presented to the ED with severe dyspnea worsening when lying flat. Past medical history has been significant for illicit methamphetamine drug use, aka crystal meth, for eight years. The patient also admits to tobacco use but denies alcohol or other illicit drugs. Past medical history also includes duodenal perforations. There was associated chest pain with the presenting orthopnea, and he claimed that his chest pain was gradually worsening over the previous week. The patient also noted swelling in his ankles and calves worsening over the last several weeks. Upon further evaluation in the emergency department, his liver enzymes and B-type natriuretic peptide (BNP) were elevated, and cardiology and gastroenterology were consulted. Dilated cardiomyopathy with acute decompensation was suspected and eventually confirmed by an echocardiography (Echo) study. The echo study revealed an ejection fraction (EF) of less than 20% by visualization. The echo was also noteworthy for severely decreased global left ventricular systolic function, severely enlarged left atrium, and moderate to severe mitral valve regurgitation. The patient was admitted into the intensive care unit (ICU) for further workup and acute management of orthopnea and acute congestive heart failure (CHF) symptoms. On the first day of ICU, an MRI of the chest was ordered and revealed severely dilated left ventricle (LV EDVi = 195 ml/m2) with severely depressed function (LV EF = 10%) and severely dilated right ventricle (RV EDVi = 165 ml/m2) with severely depressed function (RV EF = 20%). Images of the MRI can be seen in Figure 1. Upon further investigation, the patient was found to have multiple previous hospitalizations with unstable but improving liver failure. Ascites was appreciated on both the exam and imaging, but improvement was attributed to alcohol cessation by the patient with an undisclosed course of previous alcohol use disorder treatment. After remaining stable on his second day in the ICU, the patient was aggressively diuresed with IV lasix, net negative 9.7L. On hospital day three, he was downgraded to cardiac telemetry and monitored for eventual discharge after 24 hours of observation. The patient declined CAD/ischemic work-up during his stay, opting instead to continue with medical management at the time. The patient was informed he would need an implantable cardioverter-defibrillator (ICD) for primary prevention, was discharged home with a life vest in place, and was given instructions to follow up as an outpatient. Upon discharge, the patient was prescribed metoprolol succinate and dapagliflozin. Losartan was attempted but put on hold due to hyperkalemia (potassium increased from 4.0 to 5.4). He was also discharged on Torsemide, with instructions to weigh himself daily and take additional doses if his weight increased. The patient was given instructions to follow up with a heart failure specialist outpatient with any new concerns and to return to the emergency department if symptoms significantly worsened. Regarding his long-standing crystal meth use, an addiction specialist was offered, but the patient ultimately declined resources and referrals for substance abuse treatment.

## Discussion

Amphetamine-induced cardiomyopathy is a rare but severe cardiac disorder caused by the long-term use of amphetamines. A study published in the Journal of Cardiology Cases examined the case of a 37-year-old man who developed dilated cardiomyopathy due to chronic amphetamine use. The study highlighted the importance of early detection and cessation of drug use in preventing further damage to the heart [6]. Another case report published described a patient with severe amphetamine-induced cardiomyopathy who was successfully treated with heart transplantation. This study emphasized once more the critical role of early intervention and appropriate management in improving patient outcomes [7].

While the exact mechanisms underlying amphetamine-induced cardiomyopathy are not fully understood, several possible pathways have been proposed, including oxidative stress, mitochondrial dysfunction, and neurohormonal activation. Recent research has shown that the incidence of amphetamine-induced cardiomyopathy appears to be increasing, likely due to the increasing availability and use of amphetamines, particularly among young adults. In addition, there is growing evidence that the use of amphetamines in combination with other drugs, such as cocaine or opioids, may further increase the risk of developing cardiomyopathy.

Despite the wealth of literature on this topic, there are still many unanswered questions regarding the optimal management and treatment of patients with amphetamine-induced cardiomyopathy. Some studies have suggested that early identification and cessation of amphetamine use may lead to improved outcomes, while others have found that the long-term prognosis for these patients remains poor, even with aggressive medical therapy.

While these case reports differ in the details of the patients' presentation, they all underscore the potential for amphetamines to cause cardiomyopathy, highlighting the importance of screening and monitoring patients who use these drugs. Additionally, they demonstrate the need for further research to better understand the underlying mechanisms of amphetamine-induced cardiomyopathy as well as potential treatment options for affected individuals.

## Conclusions

Overall, the current literature suggests that amphetamine-induced cardiomyopathy is a significant public health concern that requires further research and attention. Efforts should be focused on developing effective prevention and treatment strategies, as well as increasing awareness among healthcare providers and the general public about the risks associated with amphetamine use. The definitive diagnosis, management, and aftercare are still highly subjective and should be the focus of future standard-of-care analysis.
